# Supplementary material for: Acid enhanced zipping effect to densify MWCNT packing for multifunctional MWCNT films with ultra-high electrical conductivity
Source: Nat Commun. 2023 Jan 24;14:380. doi: 10.1038/s41467-023-36082-2 (PMC9873916; doi:10.1038/s41467-023-36082-2)
Supplement: Supplementary file 1 — Supplementary information [file 41467_2023_36082_MOESM1_ESM.pdf]

Supplementary information

Acid enhanced zipping effect to densify MWCNT packing for multifunctional MWCNT films with ultra-high electrical conductivity

Hong Wang[1,2*‡], Xu Sun[1‡], Yizhuo Wang[1], Kuncai Li[1], Jing Wang[1], Xu Dai[1], Bin Chen[1,2], Daotong Chong[1,2], Liuyang Zhang[3], Junjie Yan[1,2]

[1] State Key Laboratory of Multiphase Flow in Power Engineering & Frontier Institute of Science and Technology, Xi’an Jiaotong University, Xi’an, 710054, China

[2] School of Energy and Power Engineering, Xi’an Jiaotong University, Xi’an, 710054, China

[3] School of Mechanical Engineering, Xi’an Jiaotong University, Xi’an, 710054, China

E-mail: hong.wang@xjtu.edu.cn

[‡]These two authors contribute equally to the manuscript.

Density calculation method: The MWCNT films contained iron nanoparticles. The theoretical density of the MWCNT film was 2.08-2.11 g/cm^3 (assuming that all the iron particles were inside the carbon nanotubes, the theoretical density of MWCNT film containing 28.7 wt.% iron was 2.11 g/cm^3. Assuming that all the iron particles were on the sidewalls of the carbon nanotubes, the theoretical density of MWCNT film containing 28.7 wt.% iron was 2.08 g/cm^3).

The practical density of MWCNT films could be calculated with the equation: $\text{density} = \text{mass}/\text{volume}$. The mass of the sample was obtained with a high-precision microbalance and the volume of the sample was estimated by the following equation: volume = length x width x thickness. The practical density of CSA-MWCNT in this work was 1.92±0.11 g/cm^3. This value was larger than the theoretical density of CNTs 1.5 g/cm^3 reported in the previous works[1] due to the existence of iron nanoparticles. The relative density for CSA-MWCNT was about 91.65±0.66%.

Cross-section area calculation method: The cross-section area value was calculated with the equation: A = width of the sample x thickness of the sample.

Layer number calculation method: A number of CSA-MWCNT layers of 217 were obtained with the equation: number of the layers = thickness of CSA-MWCNT film/(average diameter of CSA-MWCNT + d-spacing) x sin60° =600 / (2.85+0.31) x 0.866.

Electrical conductivity calculation method: The electrical conductivity (σ) of the sample was calculated with the following equation: $\sigma=1/\rho= L/(R\cdot A)$, where ρ was the resistivity, L was the length of the sample between electrode 2 and electrode 3, R was the resistance of the sample between electrode 2 and electrode 3 (**Figure S18**), A was the cross-sectional area of the sample.

The cross-sectional areas were 0.0417±0.0046 mm^2, 0.0353±0.0017 mm^2, 0.0332±0.0010 mm^2, and 0.0032±0.0002 mm^2 for pristine-MWCNT films, annealed-MWCNT films, HCl-MWCNT films and CSA-MWCNT films, respectively. This cross-sectional area was pretty uniform with relatively small

error bars as shown in the following **Table S1** since the pristine-MWCNT films were compressed before used. These films had a relatively flat surface as shown in **Figure S6**. The voided area of the CNT cylinder was included in the cross-sectional area calculation.

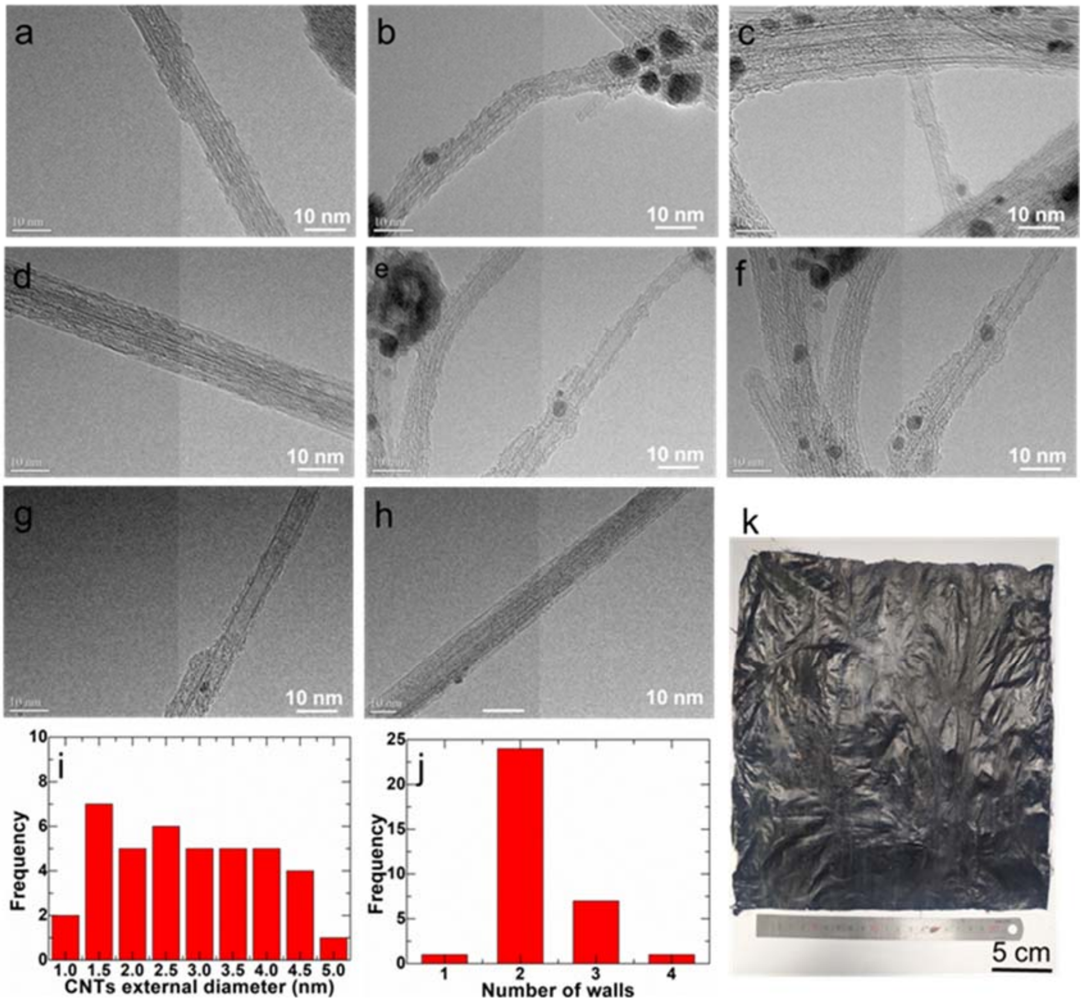

Figure S1 Transmission electron microscope (TEM) images of the MWCNT film a)-h). i) External CNT diameter distribution as determined from TEM images. j) Distribution of the number of walls per CNT as determined from TEM images. k) Optical image of the synthesized pristine-MWCNT film.

The obtained MWCNT films contained CNTs and irregular nanoparticles. Nearly 94% MWCNTs had either double walls or triple walls with a broad distribution in diameter of 1-5 nm as counted from the transmission electron microscope images shown in **Figure S1.** The diameter for most nanoparticles was in the range of 5-10 nm.

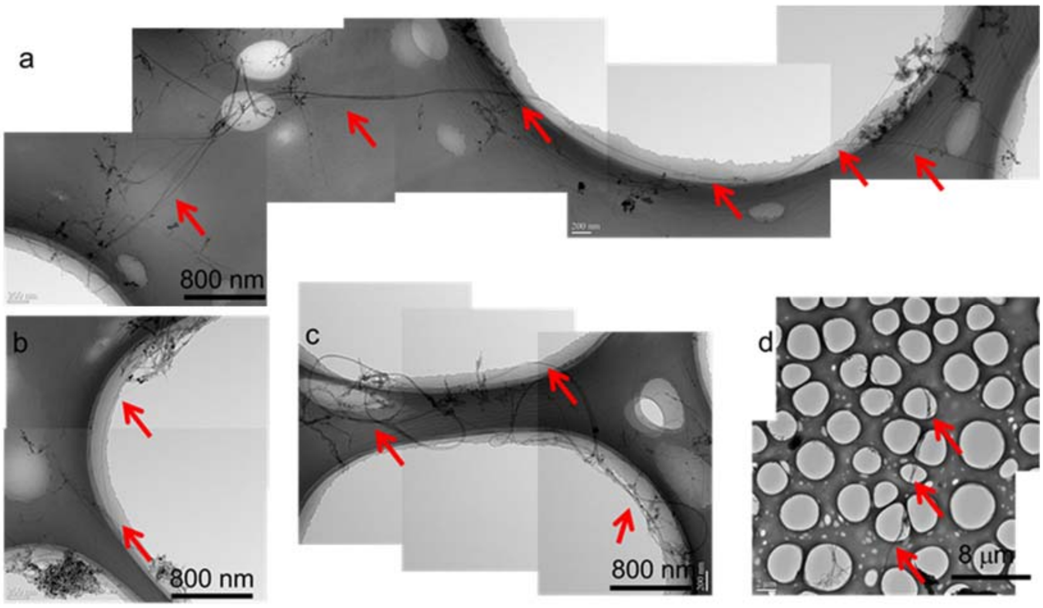

Figure S2 TEM images of MWCNTs/MWCNT bundles for the length evaluation.

It was very challenging to obtain the accurate length of CNTs synthesized with a floating catalytic chemical vapor deposition method.[2] Because the CNTs or CNT bundles growing from the same catalyst particle would be aggregated to form very complex three-dimensional networks during the growth of CNTs with the floating catalytic chemical vapor deposition method. The length of the MWCNTs/MWCNT bundles was roughly estimated to be about 20 μm as shown in the transmission electron microscope images in **Figure S2**, which was similar to the values (<20 μm) reported previously.[3, 4]

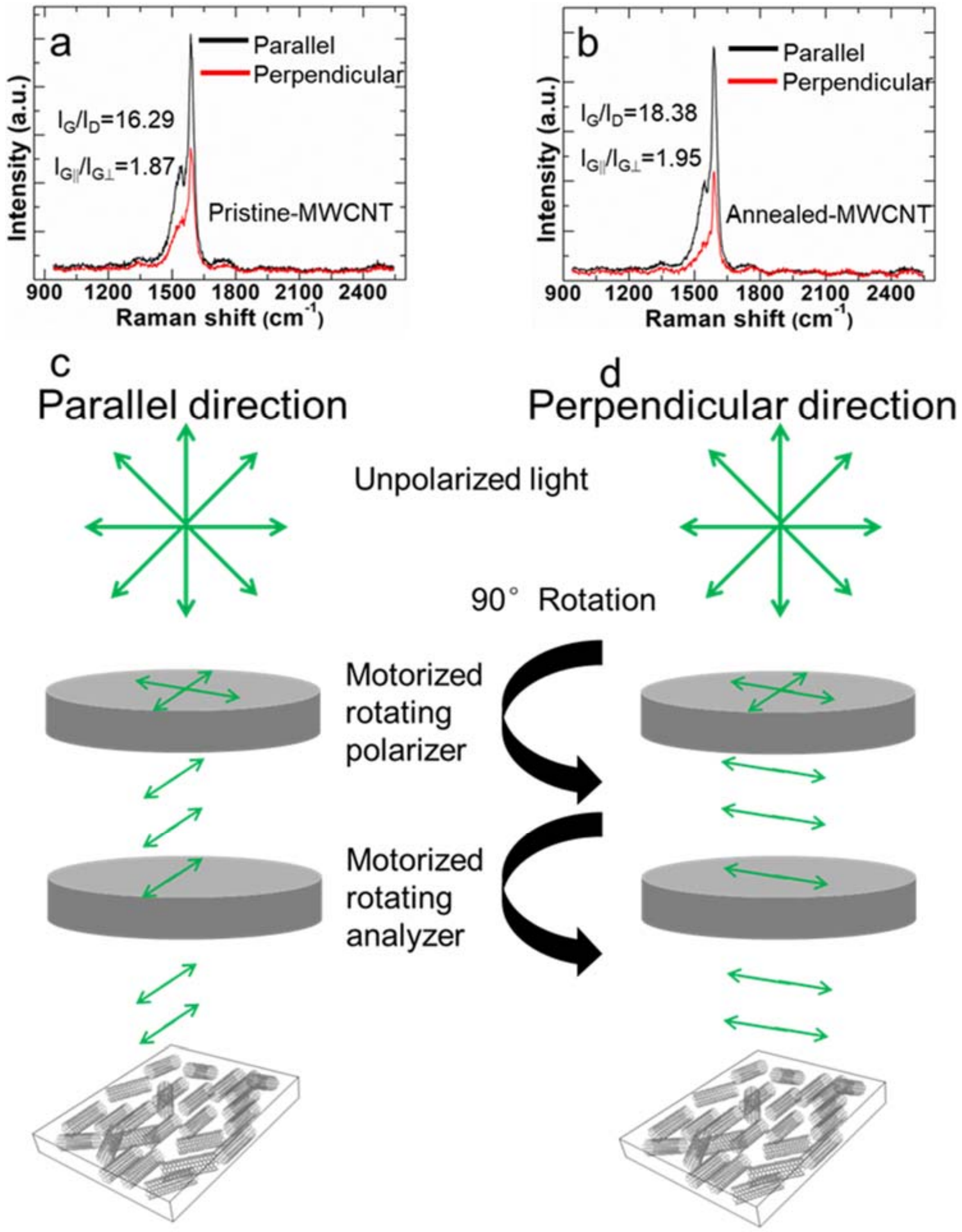

Figure S3 Raman spectra of pristine MWCNT (a), annealed-MWCNT (b).

Raman spectra indicated that the MWCNTs preferred to align along the direction parallel to the winding direction with an $I_{G\parallel}/I_{G\perp}$ ratio of 1.87 (**Figure S3**). The high I_G/I_D ratio of 16.3 also demonstrated the high quality of the synthesized MWCNT films. After annealing, the $I_{G\parallel}/I_{G\perp}$ and I_G/I_D ratios became 1.95 and 18.38, respectively.

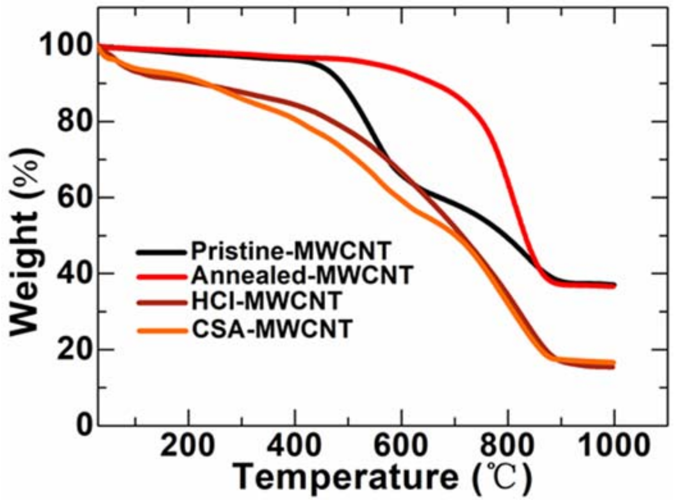

Figure S4 Thermal gravimetric curves of pristine MWCNT and annealed-MWCNT film in the air atmosphere.

Pristine-MWCNTs showed two weight losses at ~350 ^{o}C and 450 ^{o}C, respectively. The first weight loss of the pristine-MWCNTs was attributed to the degradation of amorphous carbon.[5] And the second weight loss of the pristine-MWCNTs was due to the oxidation of carbon nanotubes (CNTs).[6] While the annealed-MWCNTs had only one weight loss at 450 ^{o}C due to the oxidation of CNTs.[6] According to the distribution of the number of walls in **Figure S1**, an average diameter (D_{CNT}) was 2.85 nm.

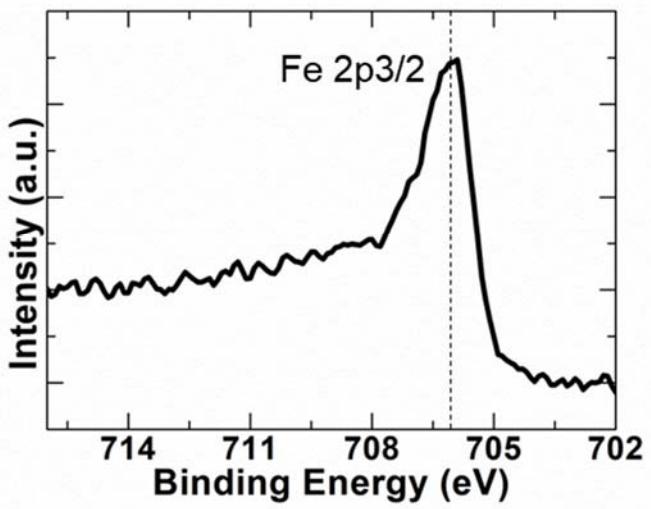

Figure S5 X-ray photoelectron spectroscopy spectrum of Fe for pristine MWCNT film.

A peak appeared at 706 eV in the X-ray photoelectron spectroscopy spectrum, which was assigned to the iron or iron carbide nanoparticles.[7] (**Figure S4**) Some of the iron nanoparticles might be oxidized in the air to reduce the electrical conductivity of the MWCNT films.[4]

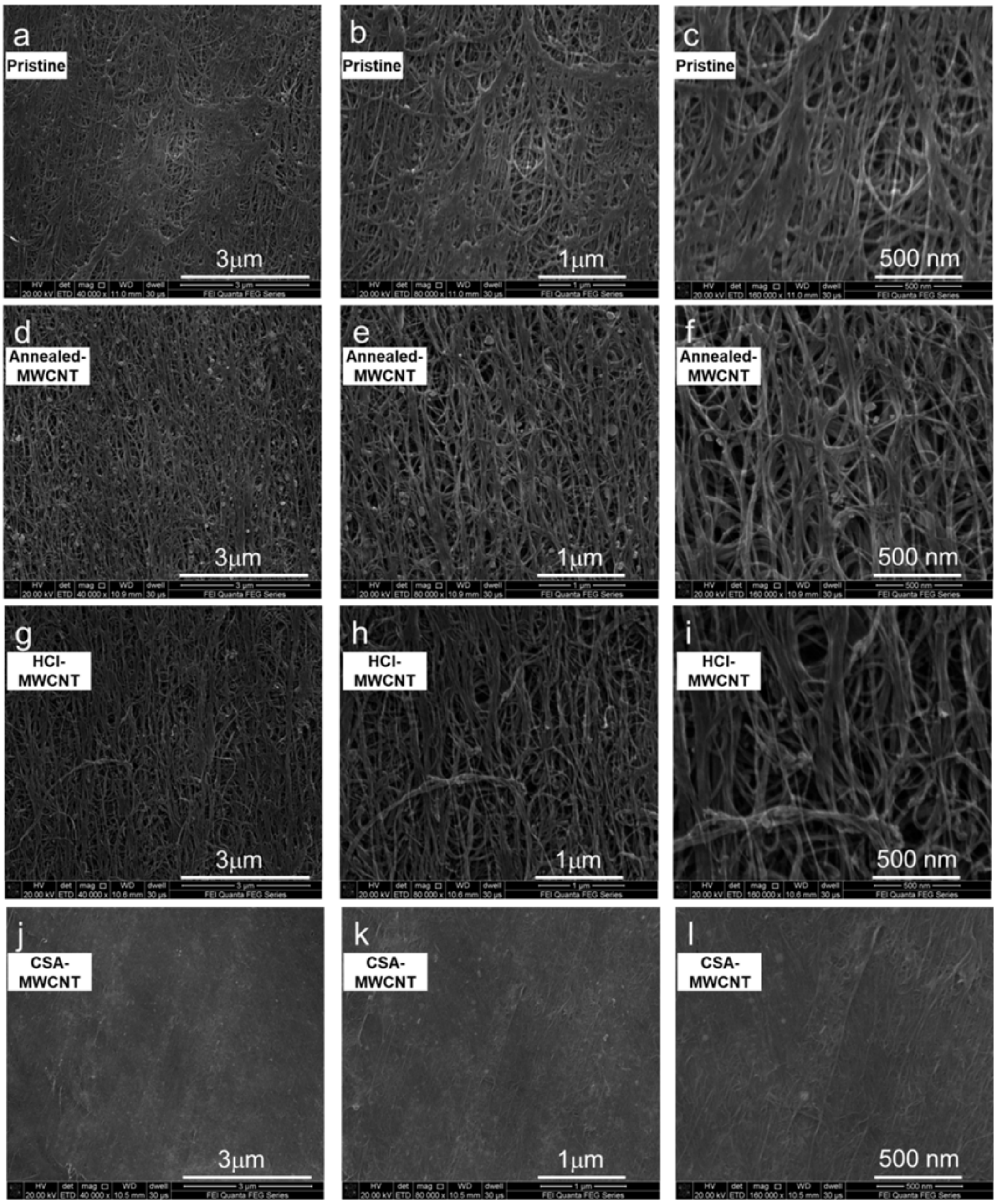

Figure S6 The scanning electron microscope (SEM) images of the morphologies of the pristine-MWCNT (a)-(c), annealed-MWCNT (d)-(f), the HCl-MWCNT (g)-(h), and CSA-MWCNT (j)-(i).

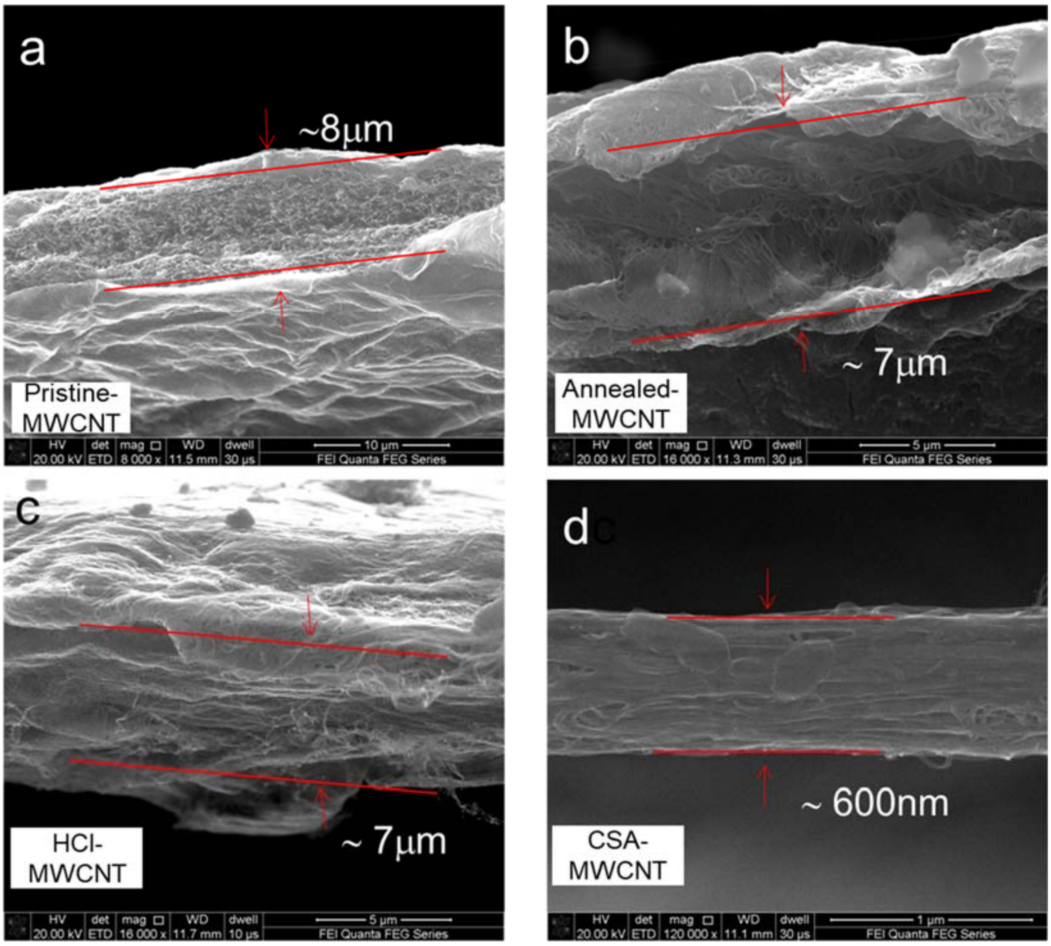

Figure S7 The scanning electron microscope (SEM) images of the cross-section of pristine-MWCNT a), annealed-MWCNT b), HCl-MWCNT c), CSA-MWCNT d).

The thicknesses of pristine-MWCNT, annealed-MWCNT, HCl-MWCNT and CSA-MWCNT were 8.34±0.92 μm, 7.07±0.35 μm, 6.64±0.19 μm, 0.64±0.05 μm, respectively, as shown in **Figure S7**. The cross-sectional areas were 0.0417±0.0046 mm^2, 0.0353±0.0017 mm^2, 0.0332±0.0010 mm^2, and 0.0032±0.0002 mm^2 for as-synthesized MWCNT films, annealed-MWCNT films, HCl-MWCNT films and CSA-MWCNT films, respectively. This cross-sectional area was pretty uniform as shown in the following **Table S1** since the pristine-MWCNT films were compressed before used. These films had a relatively flat surface as shown in **Figure S6**.

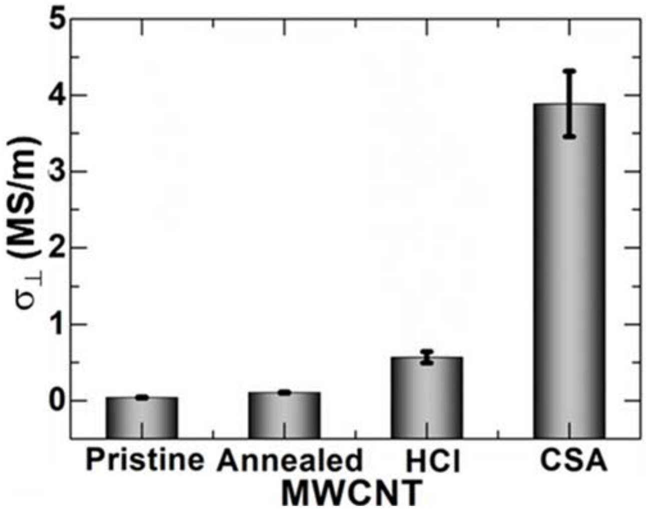

Figure S8 The $\sigma_{\perp}$ values of annealed-MWCNT, HCl-MWCNT and CSA-MWCNT

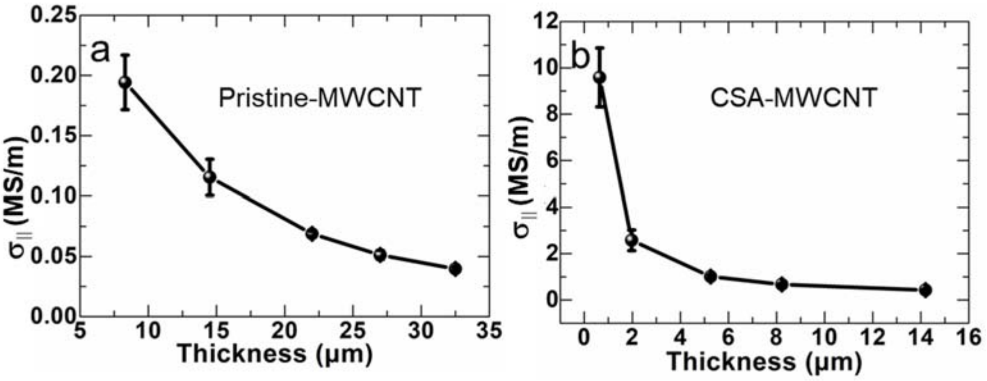

Figure S9 a) The $\sigma_{\parallel}$ of the pristine-MWCNT film as a function of the thickness. b) The $\sigma_{\parallel}$ of the CSA-MWCNT film as a function of the thickness.

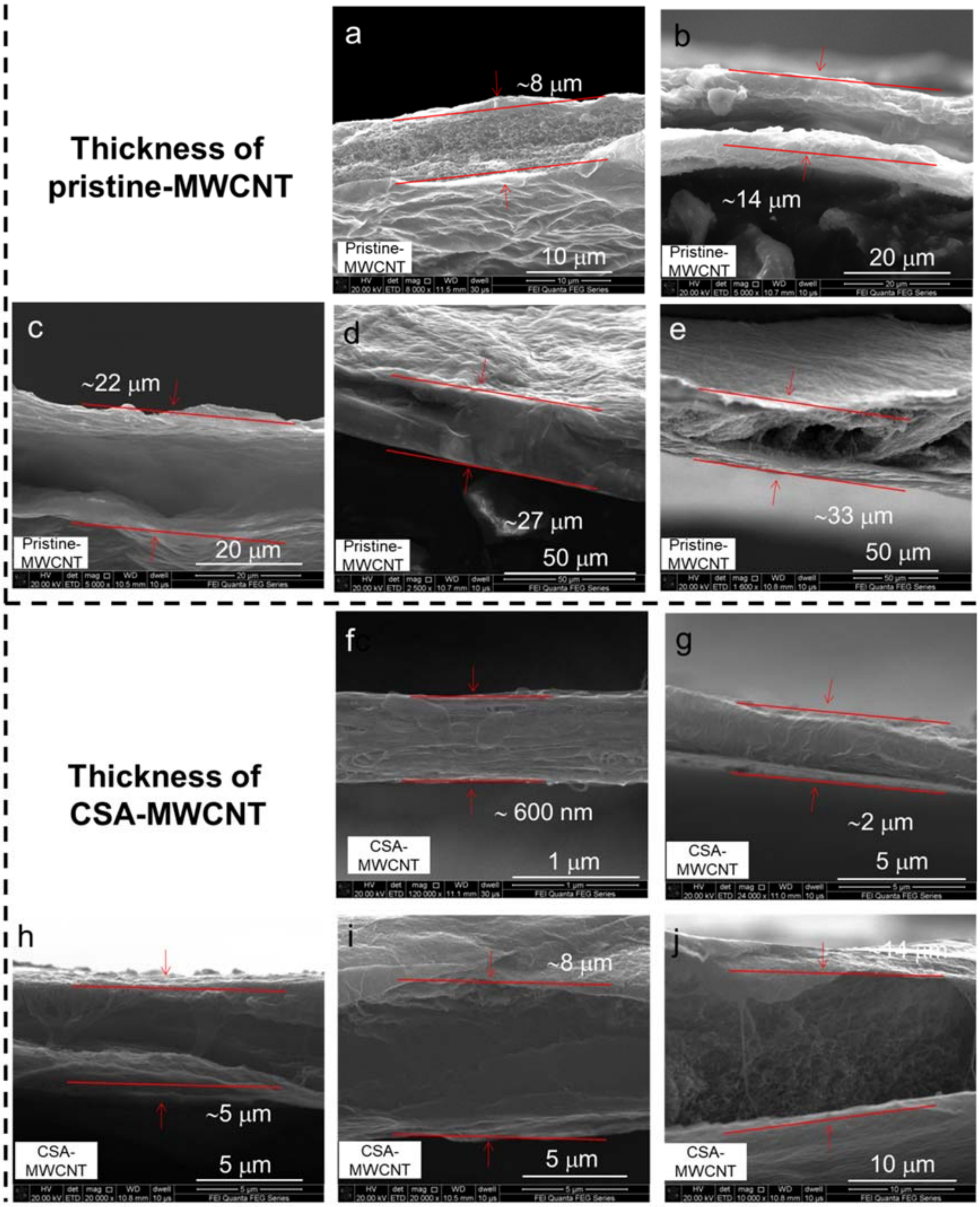

Figure S10 The scanning electron microscope (SEM) images of the cross-section of pristine-MWCNT a-d), CSA-MWCNT e-h).

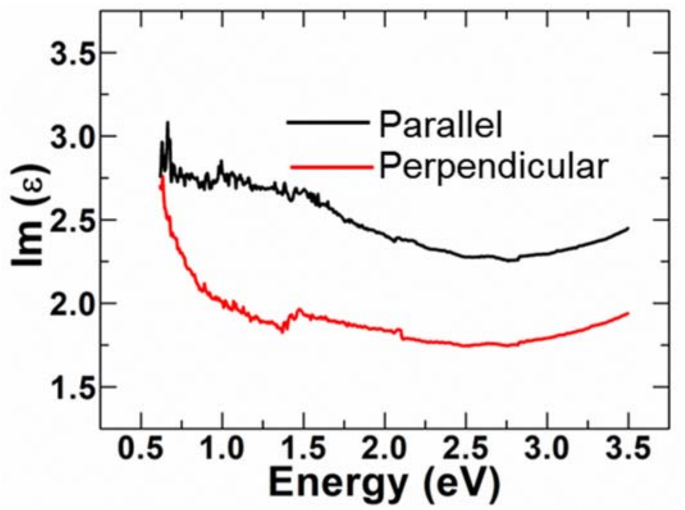

Figure S11 Dielectric functions of the CSA-MWCNT film.

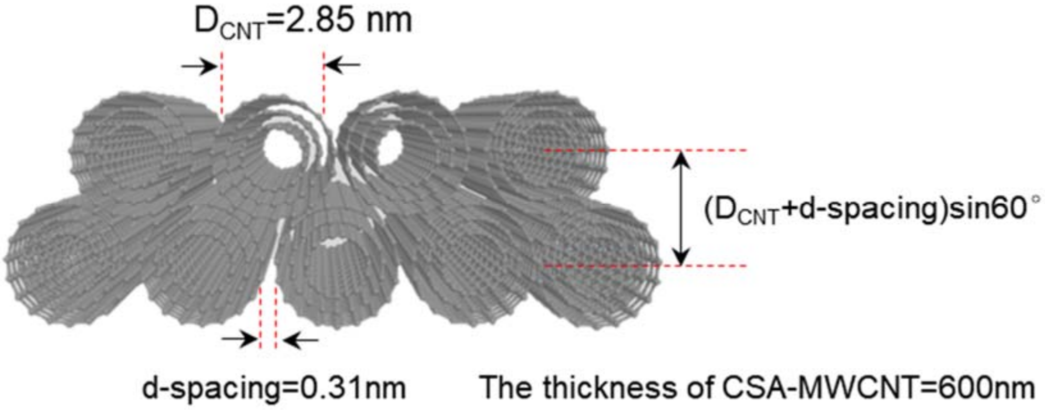

Figure S12 Illustration of MWCNT packing in the CSA-MWCNT film for layer number calculation.

According to the distribution of the number of walls in **Figure S1**, an average diameter (D_{CNT}) of 2.85 nm for MWCNTs was used in the calculation as shown in **Figure S12**.

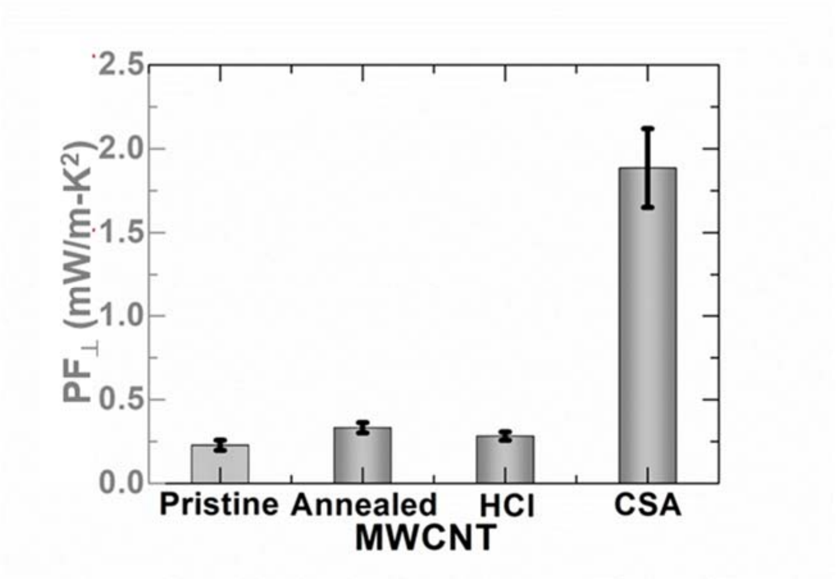

Figure S13 $PF_{\perp}$ values for pristine-MWCNT, annealed-MWCNT, HCl-MWCNT and CSA-MWCNT.

$PF_{\perp}$ values were also measured for the MWCNT films which were in the order of $PF_{\perp}$(CSA-MWCNT) > $PF_{\perp}$(HCl-MWCNT) > $PF_{\perp}$(annealed-MWCNT) > $PF_{\perp}$(pristine-MWCNT).

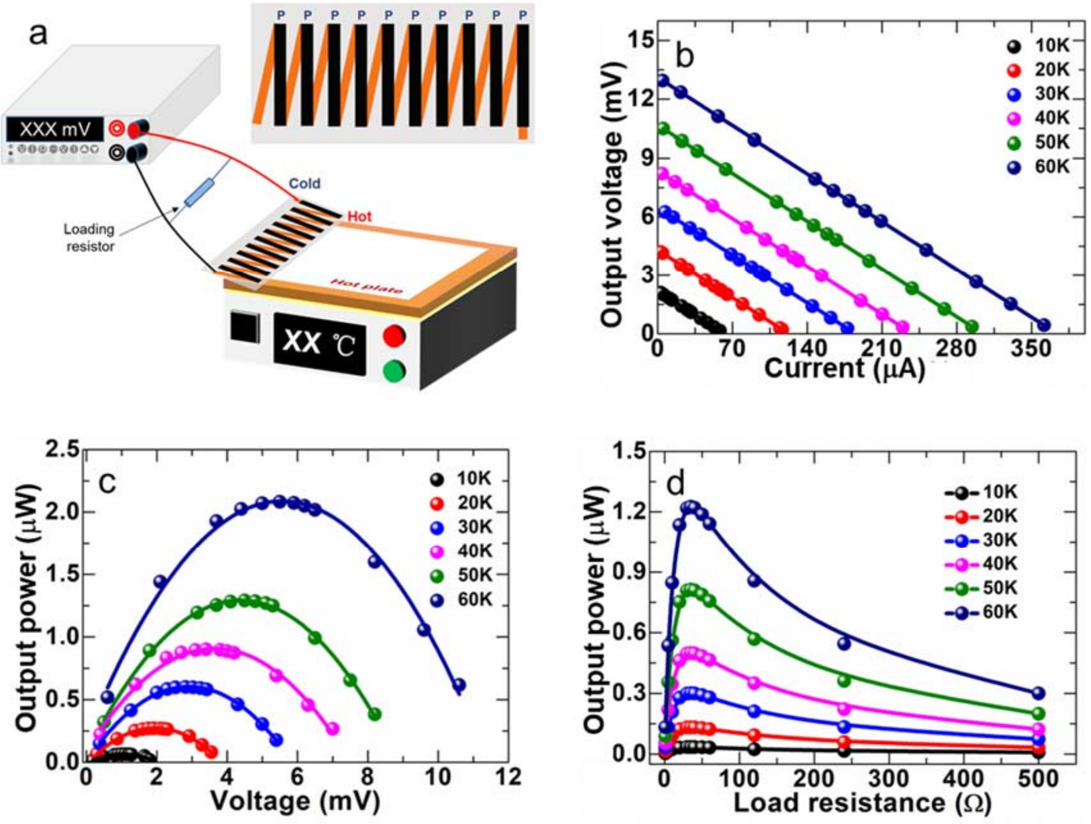

Figure S14 a) Schematic diagram of thermoelectric device testing. b) The voltage–current curves of the TE device at different temperature gradients. c) The power–voltage curves of the TE device. d) The generated power as a function of load resistance.

A thermoelectric generator (TEG) was fabricated with 10 pairs of legs to demonstrate the heat to electricity conversion ability of the obtained high $PF_{\parallel}$ CSA-MWCNT films. The p- and n-type legs were made of the CSA-MWCNT films and the copper foil with a length of 34 mm and a width of 4 mm. The p-type legs were cut with the length of the legs parallel to the winding direction.

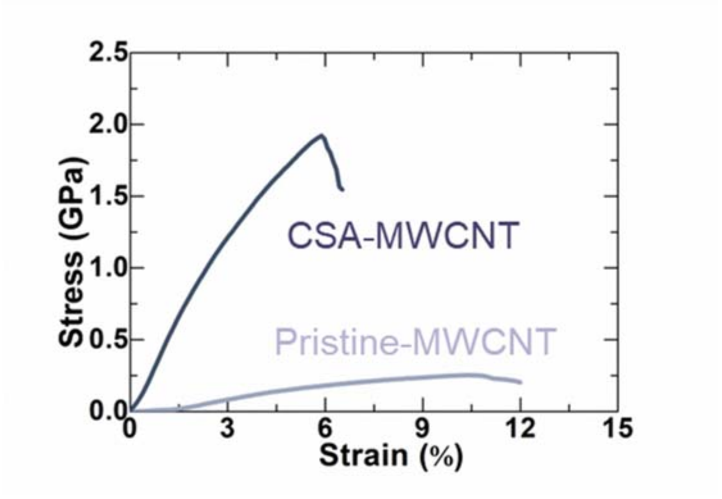

Figure S15 The stress−strain curves of pristine-MWCNT and CSA-MCNT films.

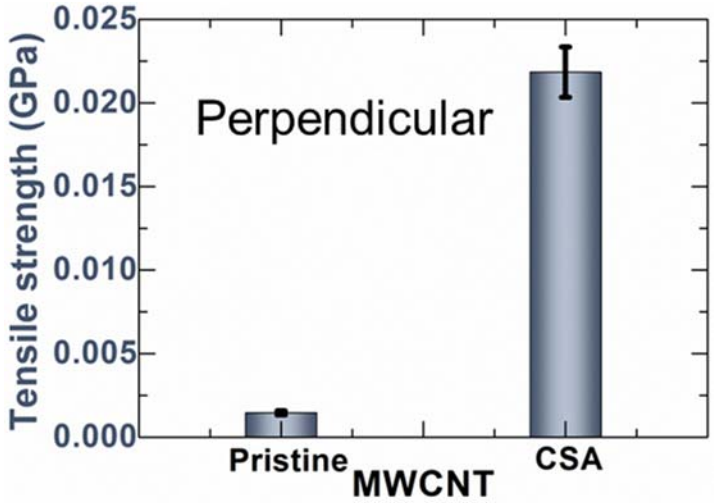

Figure S16 The tensile strength of the pristine-MWCNT film and CSA-MWCNT film in the direction perpendicular to the winding direction.

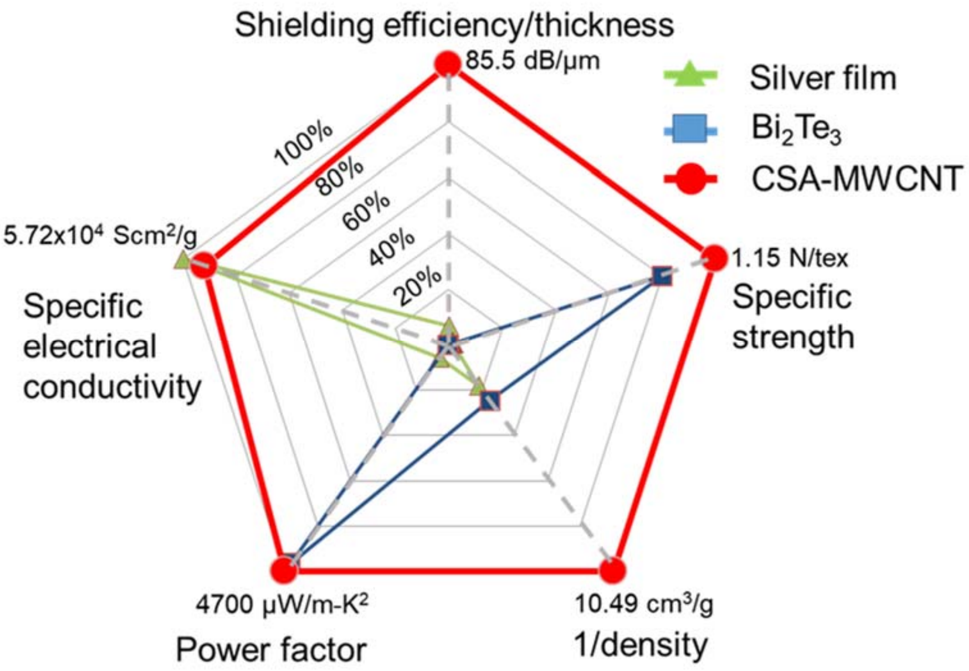

Figure S17 A radar plot showing a comparison of comprehensive performance covering specific shielding, specific electrical conductivity, power factor, 1/density and specific strength.

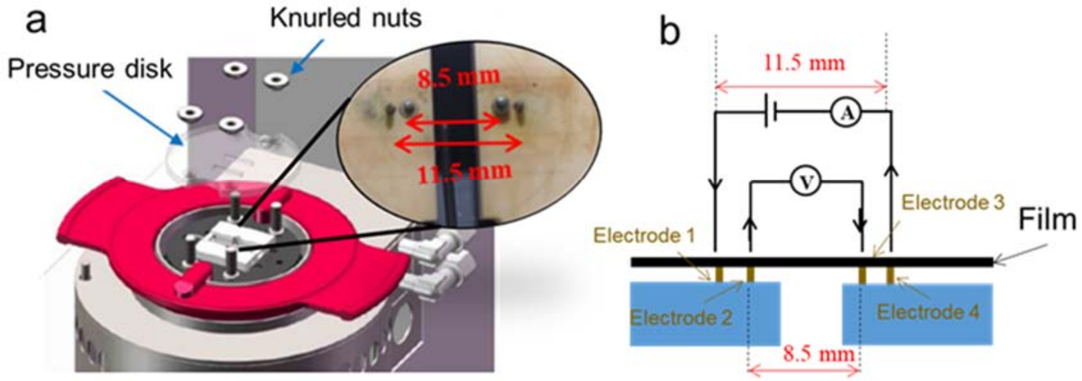

Figure S18 (a) optical image of the sample holder for the electrical conductivity measurement; (b) Illustration of the electrical circuit for the electrical conductivity measurement with a four-probe method.

The electrical conductivity of the film was measured by commercial equipment (NETZSCH SBA-458, Germany) with a four-probe method. All the measurements were performed under Ar protection at room temperature and the MWCNT film samples were cut into strips with a length of 20 mm and a width of 5 mm. The distance between the electrodes could be seen in **Figure S18**, which is about 3-8.5 mm.

The electrodes were made of rhodium which contacted directly with the samples. The pressure was applied to ensure good contact between the sample and the rhodium electrodes by a pressure disk and the knurled nuts as shown in **Figure S18**. The electrical contact resistance between the electrodes and the samples was eliminated by the four-probe method.

Table S1 The electrical conductivities and thicknesses of CNT films

**Sample**	**Thickness (μm)**	**Electrical conductivity $\sigma_{\parallel}$ (MS/m)**	**Electrical conductivity $\sigma_{\perp}$ (MS/m)**
Pristine-MWCNT	8.34±0.92	0.18±0.03	0.04±0.006
Annealed-MWCNT	7.07±0.35	0.49±0.023	0.11±0.006
HCl-MWCNT	6.64±0.19	2.33±0.24	0.56±0.07
CSA-MWCNT	0.64±0.05	9.92±1.75	3.88±0.42

Table S2 The maximum $\sigma_{\parallel}$ of CSA-MWCNT films was compared with that of CNT only and CNT composite films reported in the literature.

Sample	Electrical conductivity (MS/m)	Date
**CSA-MWCNT**	**~10**	**This work 2022**
CNT/AgNW	1.3	2019[8]
MWCNT/TCNQ	~1.02	2022[9]
CNT	0.90	2020[10]
g/gPDA/CNT	0.67	2020[10]
silver modified carbon nanotube	0.51	2020[11]
CNT	0.49	2021[12]
Pedot-Tos/a-SWCNT	0.47	2021[13]
Long m-DWCNT	0.33	2018[14]
RGO/CNT	0.28	2020[15]
Graphene/CNT	0.27	2018[16]
SWCNT	0.23	2021[17]
Graphene/CNT	0.18	2021[18]
W-CNT	~0.15	2018[19]
NDI/SWCNT	0.13	2020[20]
PEDOT:PSS/SWCNT	0.16	2021[21]
CNT/P3HT	0.1	2021[22]
PEDOT / Te / SWCNT	0.09	2020[23]
HDA-g-SWCNT	0.07	2019[24]
rGO/PDA/MWCNT	0.06	2020[25]

Table S3 SSE(CSA-MWCNT) was compared with the SSE values of state-of-the-art EMI shielding materials in the literature.

Sample	SSE (dB/μm)	SE (dB)	Thickness (μm)	Density (g/cm^3)	Date
**CSA-MWCNT**	**85.5**	**51.3**	**0.60**	**1.9**	**This work 2022**
Graphite film	72.21	27.8	0.385	2.25	2020[26]
$Ti_3C_2T_x$	50	70	1.4	-	2020[27]
$Ti_3C_2T_x$	48.93	46	0.94	4.3	2020[28]
Cu Film	41.33	49.6	1.2		2021[29]
CNT/PTA film	30	30	1	-	2020[30]
CNT film	27.67	51.2	1.85	1.39	2020[10]
MXene film	22.29	53.5	2.4	4.3	2020[28]
Ag-wrapped nanofiber membranes	22.04	55.1	2.5	1.97	2020[31]
Pristine MXene film	21.6	54	2.5	3.9	2019[32]
Cu-wrapped nanofiber membranes	21.28	53.2	2.5	1.6	2020[31]
LBL MXene-CNT	14.49	3	0.207	2.84	2018[33]
Graphene Sheet	13.92	39	2.8	2.14	2021[34]
Graphene Film	10.82	36.8	3.4	2.03	2019[35]
Graphene film	9.25	38.1	4	1.49	2020[36]
MXene/BC Film	9.25	37	4	3.17	2021[34]
MXene Film	8.40	58.4	6.95		2020[37]
Al foil	8.25	66	8	2.71	2016[38]
Annealed Ti_3CNT_x film	7.51	75.1	10	-	2020[39]
Cu foil	7	70	10	8.96	2016[40]
N-Doped MXene/HCFG/AgNW	6.65	73.2	11	-	2022[41]
MXene film	6.18	68	11	2.39	2016[40]
Graphene/ Phosphorus Film	5.88	30	5.1	-	2020[42]
Cu/graphene film	5.77	52	9	2.1	2018[43]
**Silver foil**	5.84	58.4	10	10.49	2018[44]
Crumpling MXene coating	5.2	52	10	-	2019[45]
CNT Buckypaper	4.62	23.1	5		2021[46]
RGO-CNT	3.84	57.6	15	1.45	2018[16]
MXene/PEDOT:PSS	3.79	42.1	11.1	1.94	2018[47]
Graphene Film ($CuCl_2$)	3.6	126	35	-	2021[48]
MXene/CNF paper	3.51	26	7.4	1.62	2018[49]
rGO/ Ga Film	3.3	66	20	1.33	2021[38]
CPAN NF/metal membrane	2.43	85.2	35	2.37	2018[44]
Aramid Nanofiber/ Graphene	2.29	48.2	21	1.82	2021[50]
AgNW/CNF	2.26	101	44.5	-	2020[51]
MXene film	2.05	80	39	-	2020[52]
MXene/ANF	1.29	48	37	-	2022[53]
Layered ANF - MXene/AgNW (40%)	1.14	57.3	50	1.23	2020[54]
Alternating MXene/CNF	1.13	39.6	35	1.61	2020[55]
Graphene Film	0.86	108	125	-	2021[56]

Table S4 $PF_{\parallel}$(CSA-MWCNT) was compared with the PF values of organic and inorganic films in the literature

	Sample	Electrical conductivity (MS/m)	Seebeck coefficient (μV/K)	Power factor (μW/m-K^2)	Date
Organic materials	**CSA-MWCNT**	**~10**	**~23**	**~4660**	**This work 2022**
	Oxygen plasma treatment of few-layer graphene	~0.07	~700	4500	2011[57]
	a-CNT web/BV	~0.22	−116	3103	2017[58]
	PANi/graphene-PEDOT:PSS/DWNT-PEDOT:PSS	0.19	120	2710	2016[59]
	PANi/graphene/PANi/DWCNT	~0.11	130	1825	2015[60]
	MWCNT/TCNQ	0.89	45	1800	2022[9]
	PEDOT:PSS/$CuCl_2$	5.2×10^{-6}	-18200	1700	2020[61]
	SWCNT/PEI	~0.36	−64	1500	2017[62]
	TDAE–PEDOT/CNT	7.3×10^{-4}	−1200	1050	2015[63]
	MWCNT/PEI	0.50	-45	1000	
	SWCNT/polystyrene	~0.21	61	789	2020[64]
	MWCNT/[HMIM][BF_4]	~0.11	~80	762	2022[65]
	SWCNT/FcMA	~0.27	-46.07	567.54	2021[66]
	TPETPA/SWCNT	~0.04	123.2	539.8	2018[67]
	PEDOT:PSS/SWCNT	0.17	55.6	526	2019[68]
	poly(metaTFSI)/SWCNT	0.1	70	490	2017[69]
	PEDOT:PSS/PSSH(PSSNa) coating after H_2SO_4	0.21	43.5	401	2018[70]
	SWCNT/PANI	~0.24	39.2	362	2020[71]
	PEDOT-PF_6/SWCNT	0.36	31.1	350	2019[72]
	PEDOT:PSS/H_2SO_4,NaOH drop	~0.22	39.2	334	2017[73]
Inorganic materials	Bi_2Te_3	~0.15	-180	4700	2021[74]
	single-crystalline Bi_2Te_3	~0.13	164	3400	2021[75]
	Bi_2Te_3	~0.07	222	3373	2022[76]
	Bi_2Te_3	~0.08	-180	2500	2020[77]
	Bi_2Te_3	~0.04	-198.9	1490	2021[78]
	Bi_2Te_3	~0.05	165	1460	2022[79]
	Bi_2Te_3	~0.04	184.2	1250	2021[78]
	Bi_2Te_3 pellet	~0.07	-133	1289	2019[80]

Table S5 The measured thermal conductivities at room temperature.

Sample	Thermal conductivity (W/m-K)				ZT
	1	2	3	Average	
Pristine-MWCNT	21.8	22.0	22.0	21.93±0.10	0.0143±0.0017
CSA-MWCNT	45.9	45.8	46.0	45.90±0.10	0.0304±0.0053

Table S6 The ampacity of the high $\sigma_{\parallel}$ CSA-MWCNT films was compared with that of CNT-only films/fibers in the literature

Material	Current density (A/cm^2)	Date
**CSA-MWCNT film**	**~170000**	**This work 2022**
DWCNT fiber	100000	2012[81]
CNT fiber	66000	2016[82]
DWCNT/I_2 cable	16200	2011[83]
CNT fiber	5800	2013[84]
CNT fiber	4550	2014[85]
CNT/$KAuBr_4$ yarn	3500	2019[86]

Table S7 Comparison of the specific shielding efficiency, the density, the specific strength and the specific electrical conductivity of silver film, Bi_2Te_3 film and CSA-MWCNT film.

Sample	Power factor (μW/m-K^2)	SSE (dB/μm)	Density (g/cm^3)	Specific strength (N/tex)	Specific electrical conductivity (Scm^2/g)
Ag film	~266[87]	~5.84[44]	10.49[44]	~0.016	~5.72x10^4
Bi_2Te_3 film	4700[74]	~0.027[88]	7.7	~0.922[89]	~194.8[90]
This work	~4660	85.5	~1.9	~1.15	5.26 x10^4

Table S8 Comparison of the tensile strength of the CSA-MWCNT film with CNT based fibers in the literature.

	Sample	Specific tensile strength (N/tex)	Tensile strength (GPa)	Density (g/cm^3)	Date
	**CSA-MWCNT film**	**1.15**	**2.2**	**~1.9**	**This work**
Fiber	CNT	4.08	4.48	1.1	2019[91]
	CNT	3.84	6.57	1.71	2022[92]
	Cross-linked CNT	3.7	1.7	0.45	2017[93]
	G-CNT	3.00	6.05	2.01	2022[94]
	PI-CNT	2.99	6.21	1.74	2022[95]
	CNT	2.55	5.02	1.97	2022[96]
	CNT	2.1	4.2	0.51	2021[97]
	DWCNT	1.6	2.4	1.5	2017[98]
	CNT/I_2	0.97	1.35	1.4	2013[84]
	CNT	~0.91	-	-	2021[99]
	CNT	0.89	1.33	1.49	2022[100]
	CNT/py-PDA/C	0.79	0.727	0.92	2019[101]

Table S9 Summary of the sizes of MWCNT films used in this work.

Sample	Size<br>Length x width (cm)
SEM	1x1
Raman	1x1
XRD	1x1
Thermoelectric	2x0.5
Electromagnetic interference	2.5x2.5
Current density	3x0.1
Strength	1x0.5

The optical image of the pristine-MWCNT film has been added in **Figure S1** in the supplementary information. Typically, the film had a length of 25 cm and a width of 28 cm. The sizes of MWCNT films used in this work have been summarized in **Table S9**.

Supplementary References:

1. Wang J. N., Luo X. G., Wu T., Chen Y. High-strength carbon nanotube fibre-like ribbon with high ductility and high electrical conductivity. *Nat Commun* **5**, 3848 (2014).

2. Zhou T., Niu Y., Li Z., Li H., Yong Z., Wu K., *et al.* The synergetic relationship between the length and orientation of carbon nanotubes in direct spinning of high-strength carbon nanotube fibers. *Mater Des* **203**, (2021).

3. Xu W., Chen Y., Zhan H., Wang J. N. High-Strength Carbon Nanotube Film from Improving Alignment and Densification. *Nano Lett* **16**, 946-952 (2016).

4. Zhang Q., Nam J. S., Han J., Datta S., Wei N., Ding E. X., *et al.* Large-Diameter Carbon Nanotube Transparent Conductor Overcoming Performance–Yield Tradeoff. *Adv Funct Mater* **32**, (2021).

5. Dini Y., Faure-Vincent J., Dijon J. How to overcome the electrical conductivity limitation of carbon nanotube yarns drawn from carbon nanotube arrays. *Carbon* **144**, 301-311 (2019).

6. Jung Y., Kim T., Park C. R. Effect of polymer infiltration on structure and properties of carbon nanotube yarns. *Carbon* **88**, 60-69 (2015).

7. Biesinger M. C., Payne B. P., Grosvenor A. P., Lau L. W. M., Gerson A. R., Smart R. S. C. Resolving surface chemical states in XPS analysis of first row transition metals, oxides and hydroxides: Cr, Mn, Fe, Co and Ni. *Appl Surf Sci* **257**, 2717-2730 (2011).

8. Oluwalowo A., Nguyen N., Zhang S., Park J. G., Liang R. Electrical and thermal conductivity improvement of carbon nanotube and silver composites. *Carbon* **146**, 224-231 (2019).

9. Sun X., Wang Y., Li K., Wang J., Dai X., Chong D., *et al.* Anisotropic Electrical Conductivity and Isotropic Seebeck Coefficient Feature Induced High Thermoelectric Power Factor > 1800 $\mu Wm^{-1}K^{-2}$ in MWCNT Films. *Adv Funct Mater* (2022).

10. Zou R., Liu F., Hu N., Ning H., Gong Y., Wang S., *et al.* Graphene/Graphitized Polydopamine/Carbon Nanotube All-Carbon Ternary Composite Films with Improved Mechanical Properties and Through-Plane Thermal Conductivity. *ACS Appl Mater Interfaces* **12**, 57391-57400 (2020).

11. Xia Q., Mei H., Zhang Z., Liu Y., Liu Y., Leng J. Fabrication of the silver modified carbon nanotube film/carbon fiber reinforced polymer composite for the lightning strike protection application. *Compos B Eng* **180**, (2020).

12. Zhan H., Chen Y. W., Shi Q. Q., Zhang Y., Mo R. W., Wang J. N. Highly aligned and densified carbon nanotube films with superior thermal conductivity and mechanical strength. *Carbon* **186**, 205-214 (2022).

13. Liang L., Wang X., Wang M., Liu Z., Chen G., Sun G. Flexible poly(3,4-ethylenedioxythiophene)-tosylate/SWCNT composite films with ultrahigh electrical conductivity for thermoelectric energy harvesting. *Compos Commun* **25**, (2021).

14. Wang P., Peng Z., Li M., Wang Y. Stretchable Transparent Conductive Films from Long Carbon Nanotube Metals. *Small* **14**, e1802625 (2018).

15. Liu S., Wang P., Liu C., Deng Y., Dou S., Liu Y., *et al.* Nanomanufacturing of RGO-CNT Hybrid Film for Flexible Aqueous Al-Ion Batteries. *Small* **16**, e2002856 (2020).

16. Zhou E., Xi J., Guo Y., Liu Y., Xu Z., Peng L., *et al.* Synergistic effect of graphene and carbon nanotube for high-performance electromagnetic interference shielding films. *Carbon* **133**, 316-322 (2018).

17. Wang Y., Li Q., Wang J., Li Z., Li K., Dai X., *et al.* Understanding the solvent effects on polarity switching and thermoelectric properties changing of solution-processable n-type single-walled carbon nanotube films. *Nano Energy* **93**, (2022).

18. Jia H., Kong Q.-Q., Yang X., Xie L.-J., Sun G.-H., Liang L.-L., *et al.* Dual-functional graphene/carbon nanotubes thick film: Bidirectional thermal dissipation and electromagnetic shielding. *Carbon* **171**, 329-340 (2021).

19. Yao Y., Jiang F., Yang C., Fu K. K., Hayden J., Lin C. F., *et al.* Epitaxial Welding of Carbon Nanotube Networks for Aqueous Battery Current Collectors. *ACS Nano* **12**, 5266-5273 (2018).

20. Wang Y., Chen Z., Huang H., Wang D., Liu D., Wang L. Organic radical compound and carbon nanotube composites with enhanced electrical conductivity towards high-performance p-type and n-type thermoelectric materials. *J Mater Chem A* **8**, 24675-24684 (2020).

21. Deng W., Deng L., Li Z., Zhang Y., Chen G. Synergistically Boosting Thermoelectric Performance of PEDOT:PSS/SWCNT Composites via the Ion-Exchange Effect and Promoting SWCNT Dispersion by the Ionic Liquid. *ACS Appl Mater Interfaces* **13**, 12131-12140 (2021).

22. Bash D., Cai Y., Chellappan V., Wong S. L., Yang X., Kumar P., *et al.* Multi-Fidelity High-Throughput Optimization of Electrical Conductivity in P3HT-CNT Composites. *Adv Funct Mater* **31**, (2021).

23. Yin S., Lu W., Wu R., Fan W., Guo C. Y., Chen G. Poly(3,4-ethylenedioxythiophene)/Te/Single-Walled Carbon Nanotube Composites with High Thermoelectric Performance Promoted by Electropolymerization. *ACS Appl Mater Interfaces* **12**, 3547-3553 (2020).

24. Cao R., Chen S., Wang Y., Han N., Liu H., Zhang X. Functionalized carbon nanotubes as phase change materials with enhanced thermal, electrical conductivity, light-to-thermal, and electro-to-thermal performances. *Carbon* **149**, 263-272 (2019).

25. Zou R., Liu F., Hu N., Ning H., Wang S., Huang K.*, et al.* Ultratough reduced graphene oxide composite films synergistically toughened and reinforced by polydopamine wrapped carbon nanotubes. *Carbon* **159**, 422-431 (2020).

26. Zhou T., Xu C., Liu H., Wei Q., Wang H., Zhang J.*, et al.* Second Time-Scale Synthesis of High-Quality Graphite Films by Quenching for Effective Electromagnetic Interference Shielding. *ACS Nano* **14**, 3121-3128 (2020).

27. Han M., Shuck C. E., Rakhmanov R., Parchment D., Anasori B., Koo C. M.*, et al.* Beyond Ti3C2Tx: MXenes for Electromagnetic Interference Shielding. *ACS Nano* **14**, 5008-5016 (2020).

28. Zhang J., Kong N., Uzun S., Levitt A., Seyedin S., Lynch P. A.*, et al.* Scalable Manufacturing of Free-Standing, Strong Ti3 C2 Tx MXene Films with Outstanding Conductivity. *Adv Mater* **32**, e2001093 (2020).

29. Choi H. K., Lee A., Park M., Lee D. S., Bae S., Lee S. K.*, et al.* Hierarchical Porous Film with Layer-by-Layer Assembly of 2D Copper Nanosheets for Ultimate Electromagnetic Interference Shielding. *ACS Nano* **15**, 829-839 (2021).

30. Wu G., Chen Y., Zhan H., Chen H. T., Lin J. H., Wang J. N.*, et al.* Ultrathin and flexible carbon nanotube/polymer composite films with excellent mechanical strength and electromagnetic interference shielding. *Carbon* **158**, 472-480 (2020).

31. Zeng Z., Jiang F., Yue Y., Han D., Lin L., Zhao S.*, et al.* Flexible and Ultrathin Waterproof Cellular Membranes Based on High-Conjunction Metal-Wrapped Polymer Nanofibers for Electromagnetic Interference Shielding. *Adv Mater* **32**, e1908496 (2020).

32. Chen H., Wen Y., Qi Y., Zhao Q., Qu L., Li C. Pristine Titanium Carbide MXene Films with Environmentally Stable Conductivity and Superior Mechanical Strength. *Adv Funct Mater* **30**, (2019).

33. Weng G. M., Li J., Alhabeb M., Karpovich C., Wang H., Lipton J.*, et al.* Layer-by-Layer Assembly of Cross-Functional Semi-transparent MXene-Carbon Nanotubes Composite

Films for Next-Generation Electromagnetic Interference Shielding. *Adv Funct Mater* **28**, (2018).

34. Wan S., Chen Y., Fang S., Wang S., Xu Z., Jiang L., *et al.* High-strength scalable graphene sheets by freezing stretch-induced alignment. *Nat Mater* **20**, 624-631 (2021).

35. Wan S., Chen Y., Wang Y., Li G., Wang G., Liu L., *et al.* Ultrastrong Graphene Films via Long-Chain π-Bridging. *Matter* **1**, 389-401 (2019).

36. Wei Q., Pei S., Qian X., Liu H., Liu Z., Zhang W., *et al.* Superhigh Electromagnetic Interference Shielding of Ultrathin Aligned Pristine Graphene Nanosheets Film. *Adv Mater* **32**, e1907411 (2020).

37. Lee G. S., Yun T., Kim H., Kim I. H., Choi J., Lee S. H., *et al.* Mussel Inspired Highly Aligned Ti3C2Tx MXene Film with Synergistic Enhancement of Mechanical Strength and Ambient Stability. *ACS Nano* **14**, 11722-11732 (2020).

38. Wang C., Gong Y., Cunning B. V., Lee S., Le Q., Joshi S. R., *et al.* A general approach to composites containing nonmetallic fillers and liquid gallium. *Sci Adv* **7**, (2021).

39. Iqbal A., Shahzad F., Hantanasirisakul K., Kim M. K., Kwon J., Hong J., *et al.* Anomalous absorption of electromagnetic waves by 2D transition metal carbonitride Ti3CNTx (MXene). *Science* **369**, 446-+ (2020).

40. Shahzad F., Alhabeb M., Hatter C. B., Anasori B., Man Hong S., Koo C. M., *et al.* Electromagnetic interference shielding with 2D transition metal carbides (MXenes). *Science* **353**, 1137-1140 (2016).

41. Shi Y., Xiang Z., Cai L., Pan F., Dong Y., Zhu X., *et al.* Multi-interface Assembled N-Doped MXene/HCFG/AgNW Films for Wearable Electromagnetic Shielding Devices with Multimodal Energy Conversion and Healthcare Monitoring Performances. *ACS Nano* (2022).

42. Zhou T., Ni H., Wang Y., Wu C., Zhang H., Zhang J., *et al.* Ultratough graphene-black phosphorus films. *Proc Natl Acad Sci USA* **117**, 8727-8735 (2020).

43. Wang Z., Mao B., Wang Q., Yu J., Dai J., Song R., *et al.* Ultrahigh Conductive Copper/Large Flake Size Graphene Heterostructure Thin-Film with Remarkable Electromagnetic Interference Shielding Effectiveness. *Small* **14**, e1704332 (2018).

44. Ji H., Zhao R., Zhang N., Jin C., Lu X., Wang C. Lightweight and flexible electrospun polymer nanofiber/metal nanoparticle hybrid membrane for high-performance electromagnetic interference shielding. *NPG Asia Mater* **10**, 749-760 (2018).

45. Li Y., Tian X., Gao S. P., Jing L., Li K., Yang H., *et al.* Reversible Crumpling of 2D Titanium Carbide (MXene) Nanocoatings for Stretchable Electromagnetic Shielding and Wearable Wireless Communication. *Adv Funct Mater* **30**, (2019).

46. Yang R., Gui X., Yao L., Hu Q., Yang L., Zhang H., *et al.* Ultrathin, Lightweight, and Flexible CNT Buckypaper Enhanced Using MXenes for Electromagnetic Interference Shielding. *Nanomicro Lett* **13**, 66 (2021).

47. Liu R., Miao M., Li Y., Zhang J., Cao S., Feng X. Ultrathin Biomimetic Polymeric Ti3C2T x MXene Composite Films for Electromagnetic Interference Shielding. *ACS Appl Mater Interfaces* **10**, 44787-44795 (2018).

48. Pang K., Liu X., Liu Y., Chen Y., Xu Z., Shen Y., *et al.* Highly conductive graphene film with high-temperature stability for electromagnetic interference shielding. *Carbon* **179**, 202-208 (2021).

49. Cao W. T., Chen F. F., Zhu Y. J., Zhang Y. G., Jiang Y. Y., Ma M. G., *et al.* Binary Strengthening and Toughening of MXene/Cellulose Nanofiber Composite Paper with Nacre-Inspired Structure and Superior Electromagnetic Interference Shielding Properties. *ACS Nano* **12**, 4583-4593 (2018).

50. Vu M. C., Park P. J., Bae S.-R., Kim S. Y., Kang Y.-M., Choi W. K., *et al.* Scalable ultrarobust thermoconductive nonflammable bioinspired papers of graphene nanoplatelet crosslinked aramid nanofibers for thermal management and electromagnetic shielding. *J Mater Chem A* **9**, 8527-8540 (2021).

51. Liang C., Ruan K., Zhang Y., Gu J. Multifunctional Flexible Electromagnetic Interference Shielding Silver Nanowires/Cellulose Films with Excellent Thermal Management and Joule Heating Performances. *ACS Appl Mater Interfaces* **12**, 18023-18031 (2020).

52. Liu Z., Zhang Y., Zhang H.-B., Dai Y., Liu J., Li X., *et al.* Electrically conductive aluminum ion-reinforced MXene films for efficient electromagnetic interference shielding. *J Mater Chem A* **8**, 1673-1678 (2020).

53. Wang J., Ma X., Zhou J., Du F., Teng C. Bioinspired, High-Strength, and Flexible MXene/Aramid Fiber for Electromagnetic Interference Shielding Papers with Joule Heating Performance. *ACS Nano* (2022).

54. Ma Z., Kang S., Ma J., Shao L., Zhang Y., Liu C., *et al.* Ultraflexible and Mechanically Strong Double-Layered Aramid Nanofiber-Ti3C2Tx MXene/Silver Nanowire Nanocomposite Papers for High-Performance Electromagnetic Interference Shielding. *ACS Nano* **14**, 8368-8382 (2020).

55. Zhou B., Zhang Z., Li Y., Han G., Feng Y., Wang B., *et al.* Flexible, Robust, and

Multifunctional Electromagnetic Interference Shielding Film with Alternating Cellulose Nanofiber and MXene Layers. *ACS Appl Mater Interfaces* **12**, 4895-4905 (2020).

56. Mirkhani S. A., Iqbal A., Kwon T., Chae A., Kim D., Kim H., *et al.* Reduction of Electrochemically Exfoliated Graphene Films for High-Performance Electromagnetic Interference Shielding. *ACS Appl Mater Interfaces* **13**, 15827-15836 (2021).

57. Xiao N., Dong X., Song L., Liu D., Tay Y., Wu S., *et al.* Enhanced thermopower of graphene films with oxygen plasma treatment. *ACS Nano* **5**, 2749-2755 (2011).

58. An C. J., Kang Y. H., Song H., Jeong Y., Cho S. Y. High-performance flexible thermoelectric generator by control of electronic structure of directly spun carbon nanotube webs with various molecular dopants. *J Mater Chem A* **5**, 15631-15639 (2017).

59. Cho C., Wallace K. L., Tzeng P., Hsu J.-H., Yu C., Grunlan J. C. Outstanding Low Temperature Thermoelectric Power Factor from Completely Organic Thin Films Enabled by Multidimensional Conjugated Nanomaterials. *Adv Energy Mater* **6**, (2016).

60. Cho C., Stevens B., Hsu J. H., Bureau R., Hagen D. A., Regev O., *et al.* Completely organic multilayer thin film with thermoelectric power factor rivaling inorganic tellurides. *Adv Mater* **27**, 2996-3001 (2015).

61. Kim B., Hwang J. U., Kim E. Chloride transport in conductive polymer films for an n-type thermoelectric platform. *Energy Environ Sci* **13**, 859-867 (2020).

62. Zhou W., Fan Q., Zhang Q., Cai L., Li K., Gu X., *et al.* High-performance and compact-designed flexible thermoelectric modules enabled by a reticulate carbon nanotube architecture. *Nat Commun* **8**, 14886 (2017).

63. Wang H., Hsu J.-H., Yi S.-I., Kim S. L., Choi K., Yang G., *et al.* Thermally Driven Large N-Type Voltage Responses from Hybrids of Carbon Nanotubes and Poly(3,4-ethylenedioxythiophene) with Tetrakis(dimethylamino) ethylene. *Adv Mater* **27**, 6855-6861 (2015).

64. Suemori K., Uemura S. High thermoelectric performance of post mechanical treated carbon nanotube films with polystyrene binder. *Phys Rev Lett* **116**, (2020).

65. Jung J., Hyun Suh E., Jeong Y., Yun D.-J., Chan Park S., Gyu Oh J., *et al.* Ionic-liquid doping of carbon nanotubes with [HMIM][BF4] for flexible thermoelectric generators. *Chem Eng J* **438**, (2022).

66. Nie X., Mao X., Li X., Wu J., Liu Y., Li B., *et al.* Combined effect of N-methyl pyrrolidone and ferrocene derivatives on thermoelectric performance of n-type single-wall carbon nanotube-based composites. *Chem Eng J* **421**, (2021).

67. Kiefer D., Giovannitti A., Sun H., Biskup T., Hofmann A., Koopmans M.*, et al.* Enhanced n-Doping Efficiency of a Naphthalenediimide-Based Copolymer through Polar Side Chains for Organic Thermoelectrics. *ACS Energy Lett* **3**, 278-285 (2018).

68. Liu S., Li H., He C. Simultaneous enhancement of electrical conductivity and seebeck coefficient in organic thermoelectric SWNT/PEDOT:PSS nanocomposites. *Carbon* **149**, 25-32 (2019).

69. Nakano M., Nakashima T., Kawai T., Nonoguchi Y. Synergistic Impacts of Electrolyte Adsorption on the Thermoelectric Properties of Single-Walled Carbon Nanotubes. *Small* **13**, (2017).

70. Guan X., Cheng H., Ouyang J. Significant enhancement in the Seebeck coefficient and power factor of thermoelectric polymers by the Soret effect of polyelectrolytes. *J Mater Chem A* **6**, 19347-19352 (2018).

71. Li P., Zhao Y., Li H., Liu S., Liang Y., Cheng X.*, et al.* Facile green strategy for improving thermoelectric performance of carbon nanotube/polyaniline composites by ethanol treatment. *Compos Sci Technol* **189**, (2020).

72. Fan W., Liang L., Zhang B., Guo C.-Y., Chen G. PEDOT thermoelectric composites with excellent power factors prepared by 3-phase interfacial electropolymerization and carbon nanotube chemical doping. *J Mater Chem A* **7**, 13687-13694 (2019).

73. Fan Z., Li P., Du D., Ouyang J. Significantly Enhanced Thermoelectric Properties of PEDOT:PSS Films through Sequential Post-Treatments with Common Acids and Bases. *Adv Energy Mater* **7**, (2017).

74. Zhang M., Liu W., Zhang C., Xie S., Li Z., Hua F.*, et al.* Identifying the Manipulation of Individual Atomic-Scale Defects for Boosting Thermoelectric Performances in Artificially Controlled Bi(2)Te(3) Films. *ACS Nano* **15**, 5706-5714 (2021).

75. Fu L., Park K., Kim S.-I., Kim B., Song H. Y., Choi W.*, et al.* High-Performance Bismuth Antimony Telluride Thermoelectric Membrane on Curved and Flexible Supports. *ACS Energy Lett* **6**, 2378-2385 (2021).

76. Lou L. Y., Yang J., Zhu Y. K., Liang H., Zhang Y. X., Feng J.*, et al.* Tunable Electrical Conductivity and Simultaneously Enhanced Thermoelectric and Mechanical Properties in n-type Bi(2) Te(3). *Adv Sci* **9**, e2203250 (2022).

77. Bao D., Chen J., Yu Y., Liu W., Huang L., Han G.*, et al.* Texture-dependent thermoelectric properties of nano-structured Bi2Te3. *Chem Eng J* **388**, (2020).

78. Qin D., Pan F., Zhou J., Xu Z., Deng Y. High ZT and performance controllable thermoelectric devices based on electrically gated bismuth telluride thin films. *Nano Energy* **89**, (2021).

79. Ao D. W., Liu W. D., Chen Y. X., Wei M., Jabar B., Li F., *et al.* Novel Thermal Diffusion Temperature Engineering Leading to High Thermoelectric Performance in Bi(2) Te(3) -Based Flexible Thin-Films. *Adv Sci* **9**, e2103547 (2022).

80. Wang Y., Liu W. D., Gao H., Wang L. J., Li M., Shi X. L., *et al.* High Porosity in Nanostructured n-Type Bi(2)Te(3) Obtaining Ultralow Lattice Thermal Conductivity. *ACS Appl Mater Interfaces* **11**, 31237-31244 (2019).

81. Song L., Toth G., Wei J., Liu Z., Gao W., Ci L., *et al.* Sharp burnout failure observed in high current-carrying double-walled carbon nanotube fibers. *Nanotechnology* **23**, 015703 (2012).

82. Liu P., Hu D. C. M., Tran T. Q., Jewell D., Duong H. M. Electrical property enhancement of carbon nanotube fibers from post treatments. *Colloids Surf A Physicochem Eng Asp* **509**, 384-389 (2016).

83. Zhao Y., Wei J., Vajtai R., Ajayan P. M., Barrera E. V. Iodine doped carbon nanotube cables exceeding specific electrical conductivity of metals. *Sci Rep* **1**, 83 (2011).

84. Behabtu N., Young C. C., Tsentalovich D. E., Kleinerman O., Wang X., Ma A. W., *et al.* Strong, light, multifunctional fibers of carbon nanotubes with ultrahigh conductivity. *Science* **339**, 182-186 (2013).

85. Wang X., Behabtu N., Young C. C., Tsentalovich D. E., Pasquali M., Kono J. High-Ampacity Power Cables of Tightly-Packed and Aligned Carbon Nanotubes. *Adv Funct Mater* **24**, 3241-3249 (2014).

86. Soule K. J., Lawlor C. C., Bucossi A. R., Cress C. D., Puchades I., Landi B. J. Sustaining Enhanced Electrical Conductivity in KAuBr4-Doped Carbon Nanotube Wires at High Current Densities. *ACS Appl Nano Mater* **2**, 7340-7349 (2019).

87. Ankireddy K., Menon A. K., Iezzi B., Yee S. K., Losego M. D., Jur J. S. Electrical Conductivity, Thermal Behavior, and Seebeck Coefficient of Conductive Films for Printed Thermoelectric Energy Harvesting Systems. *J Electron Mater* **45**, 5561-5569 (2016).

88. Chen J., Liang X., Quan B., Yang Z., Du Y., Ji G. 3D Flake-Like Bi2
Te3
with Outstanding Lightweight Electromagnetic Wave Absorption Feature. *P Part Part Syst Char* **35**, (2018).

89. Tong Y., Yi F., Liu L., Zhai P., Zhang Q. Molecular dynamics study of mechanical properties of bismuth telluride nanofilm. *Physica B Condens Matter* **405**, 3190-3194 (2010).

90. Ren C., Zhu W., Zhou J., Wu X., Deng Y. Electromigration reliability and activation energy of Bi2Te3 thermoelectric film. *Appl Phys Lett* **120**, (2022).

91. Lee J., Lee D. M., Jung Y., Park J., Lee H. S., Kim Y. K., *et al.* Direct spinning and densification method for high-performance carbon nanotube fibers. *Nat Commun* **10**, 2962 (2019).

92. Lee D., Kim S. G., Hong S., Madrona C., Oh Y., Park M., *et al.* Ultrahigh strength, modulus, and conductivity of graphitic fibers by macromolecular coalescence. *Sci Adv* **8**, eabn0939 (2022).

93. Park O.-K., Choi H., Jeong H., Jung Y., Yu J., Lee J. K., *et al.* High-modulus and strength carbon nanotube fibers using molecular cross-linking. *Carbon* **118**, 413-421 (2017).

94. Kim S. G., Heo S. J., Kim J. G., Kim S. O., Lee D., Kim M., *et al.* Ultrastrong Hybrid Fibers with Tunable Macromolecular Interfaces of Graphene Oxide and Carbon Nanotube for Multifunctional Applications. *Sci Adv* **9**, e2203008 (2022).

95. Kim S. G., Heo S. J., Kim S., Kim J., Kim S. O., Lee D., *et al.* Ultrahigh strength and modulus of polyimide-carbon nanotube based carbon and graphitic fibers with superior electrical and thermal conductivities for advanced composite applications. *Compos B Eng* **247**, (2022).

96. Kim S. G., Choi G. M., Jeong H. D., Lee D., Kim S., Ryu K.-H., *et al.* Hierarchical structure control in solution spinning for strong and multifunctional carbon nanotube fibers. *Carbon* **196**, 59-69 (2022).

97. Taylor L. W., Dewey O. S., Headrick R. J., Komatsu N., Peraca N. M., Wehmeyer G., *et al.* Improved properties, increased production, and the path to broad adoption of carbon nanotube fibers. *Carbon* **171**, 689-694 (2021).

98. Tsentalovich D. E., Headrick R. J., Mirri F., Hao J., Behabtu N., Young C. C., *et al.* Influence of Carbon Nanotube Characteristics on Macroscopic Fiber Properties. *ACS Appl Mater Interfaces* **9**, 36189-36198 (2017).

99. Kim Y.-K., Kim Y.-J., Park J., Han S. W., Kim S. M. Purification effect of carbon nanotube fibers on their surface modification to develop a high-performance and multifunctional nanocomposite fiber. *Carbon* **173**, 376-383 (2021).

100. Liu F., Wang Q., Zhai G., Xiang H., Zhou J., Jia C., *et al.* Continuously processing waste

lignin into high-value carbon nanotube fibers. *Nat Commun* **13**, 5755 (2022).

101. Zhang S., Hao A., Nguyen N., Oluwalowo A., Liu Z., Dessureault Y., *et al.* Carbon nanotube/carbon composite fiber with improved strength and electrical conductivity via interface engineering. *Carbon* **144**, 628-638 (2019).
