# Supplementary material for: Acid enhanced zipping effect to densify MWCNT packing for multifunctional MWCNT films with ultra-high electrical conductivity
Source: Nat Commun. 2023 Jan 24;14:380. doi: 10.1038/s41467-023-36082-2 (PMC9873916; doi:10.1038/s41467-023-36082-2)
Supplement: Supplementary file 3 — Description of Additional Supplementary Files [file 41467_2023_36082_MOESM3_ESM.docx]

File Name: Supplementary Video 1

Description: A piece of CSA-MWCNT film successfully shielded the electromagnetic waves generated by a Tesla coil of 50 Hz

File Name: Supplementary Video 2

Description: White light produced at a current of 0.5 A

File Name: Supplementary Video 3

Description: A piece of CSA-MWCNT film with a width of 9 mm and thickness of ~1 μm pulls up a water bottle of ~1.9 kg
